# An alternative, non-intrauterine hypothesis, based on maternal mitochondrial oocyte inheritance, to explain inconsistent findings of birth weight on (breast) cancer risk

**DOI:** 10.1038/sj.bjc.6600978

**Published:** 2003-05-27

**Authors:** P A H van Noord

**Affiliations:** 1 Julius Center University Medical Center Utrecht (UMCU), P.O. Box 85500, 3508 GA Utrecht, The Netherlands

**Sir**

With great interest, I read the different results found for birth weight and birth length on adult breast cancer risk reported by Vatten (2002) and Sanderson (2002). Both articles referred to a hypothesis on early through postconception life events and breast cancer risk (Trichopoulos, 1990). In addition, Vatten found no relation between placenta weight and breast cancer risk. I wonder whether an alternative explanation, which relates to the mother's age at birth, would have given more consistent results across the two studies. Several hypotheses on the role of postconception early-life events and adult disease risk seem equally compatible with a preconception hypothesis on qualities of the oocyte reflected by maternal age at conception/birth (van Noord, 2002). I developed this hypothesis in an attempt to unify de Waard's early-life hypothesis (de Waard, 1988) with Trichopoulos' (1990) and Barker's (1993) intrauterine hypotheses. All three hypotheses rely heavily on an adequate energy production for human growth, essential for any maturation or development.

The viability of oocytes seems to be codetermined by the quality of their mitochondria and the related production of energy/ATP, which is known to decline with age and ageing (Papa, 1996; Ozawa, 1997). The importance of viable mitochondria at conception is illustrated by *in vitro* fertilisations where successful implantation, among biologically older women, became possible again when their own less viable oocytes were coinjected upon conception with mitochondria from a younger mother (Barritt, 2001). Repopulation of cells by heteroplasmic mitochondrial DNA (mtDNA) and relations with cell ‘ageing’ was observed before (King, 1989).

In the hypothesis, I propose that maternal age at birth (and conception) actually reflects the quality of the mitochondria with which an individual begins his or her life. This preconceptional mitochondrial hypothesis can also account for the opposing effects on carcinogenesis of caloric restriction and the role of free radicals that spill from the Krebs's cycle, as both relate to the proper functioning of the energy production by the mitochondria (Beckman, 1998; Merry, 2002). This also may be linked to the phenomena of selective cell death like spontaneous abortion early and apoptosis later in life (Hengartner, 2000).

The role of mitochondria is relevant to the interpretation of the results of Vatten because they play such an essential role in cell metabolism through their production of energy/ATP. The correlation of maternal age at birth with mitochondrial viability would link a subcellular phenomena to those observable at the level of populations as can be studied by epidemiology. Several (breast) cancer risk factors in adult life such as adult height, age at first childbirth, BMI, age at natural menopause and breast dysplasia, show some correlation with maternal age at birth (van Noord, 2001; van Noord, 2002).

Maternal age at birth may represent not just the biological age of the maternal oocyte, but may also reflect the age of the mother's mother (the maternal grandmother), where the initial meiotic cleavage of such an oocyte occurred (Warburton, 1997). From this meiosis-I time onwards, the mtDNA would have started to accumulate mutations even in the resting nondividing oocyte, since circular mtDNA as opposed to nucleair DNA lacks protection by histones or DNA repair (Attardi, 1981; Borst, 1981; Wallace, 1989).

There are reasons to believe that all these periconceptional phenomena represent purely maternal-, and not paternal-inherited epigenetic aspects that operate well before another maternal factor, the postimplantation placenta-dependent intrauterine environment, of Trichopoulos' hypothesis can play a role. This is evident by the lack of correlation between placenta weight and breast cancer risk reported by Vatten (2002). The placenta as a predominantly foetal organ (Susser, 1982) depends for its growth on paternal genes active in the blastocyst, especially the IGF-2 gene, which becomes expressed only around nidation (Ferguson, 2001). Before this time the paternal IGF-2 gene is silenced by imprinting an epigenetic process that starts immediately after conception and is finished before the first mitosis of the zygote (Oudejans, 1997). Thus a placenta can only have played a role long *after* imprinting and gastrulation, when by then the most crucial first cell cycles have already taken place (Wolpert, 1991). All these processes from conception, transport in the oviduct until nidation and until placentation, cover at least the first five cell divisions, and have by then resulted in at least 32 (2^5^) cells that cover 10% of all the 50 divisions an embryonic cell can make in a human lifetime (Hayflick, 1961). These first divisions and this initial cell mass were purely dependent on preconception maternal factors that during follicle growth have determined the nutrient and energy stores in the ooplasm and the number of mitochondria in the oocyte before ovulation. Thereafter, the viability of and the number of mitochondria per oocyte are crucial for ATP production required for all these early, highly energy-dependent processes (Wilson, 1992) as well as all growth after placentation. In addition to sufficient calories (glucose), there is evidence for a role of other essential nutrients on the proper functioning of the respiratory enzymes of the mitochondrial membrane-bound Kreb's cycle. Such nutrients can mend deficiencies, either in specific diseases (Eleff, 1984) or enzyme deficiencies accumulating with age(ing) (Ames, 2002). Suboptimal energy production by less viable mitochondria may, for example, affect the correct functioning of the cell spindle required for proper chromosome separation during these first five cell divisions, resulting either in abortion or when the fertilized egg survives, aneuploidy in the foetus and related birth defects.

The increases in aneuploidy run parallel to the decline in fecundity in biologically older mothers (van Noord-Zaadstra, 1999). In the surviving foetuses carrying aneuploidy and/or a higher propensity to aneuploidy, a high maternal age is reflected in an increased risk of several cancers in their offspring (Abelin, 1965). From this perspective of defective growth and development, it is interesting that aneuploidy is also an essential trait of cancer cells, as described already more than hundred years ago by Boveri (Deusberg, 1998). Also in adult cells and tissues, mitochondria play a pivoting role in proper cell function. Improper functioning cells can be eliminated selectively by apoptosis in which mitochondria also play a cruxial role (Hengartner, 2000) or when escaping apoptosis, show signs of aneuploidy and become cancerous (Deusberg, 1998).

Birth weight and length at birth have not only been linked to IGF-2 but also to IGF-1. A role of the IGF-1 axis on breast cancer, however, has only been found for premenopausal breast cancers (Bruning, 1995). Since birth weight effects on breast cancer risk were described both in pre- as well as in postmenopausal breast cancers, IGF-1 levels seem a less likely candidate to explain the inconsistent findings on birth weight and breast cancer risk in the literature. Hence, my oocyte hypothesis on how cancer risk and birth weight may both be maternally codetermined, not only by late intrauterine factors, but also by early epigenetic transmitted mitochondria, preceding conception and through their influence on proper energy production, may reconcile discrepant findings between birth weight and cancer risk.

Reanalysis of the data by Vatten and possibly also the data of Sanderson, using maternal age at birth instead of birth weight could test whether and, if so, to what extent, this preconception hypothesis on the role of mitochondria required for optimal cell function could better explain subsequent cancer risks.
